# Establishment of Long-Term Primary Cortical Neuronal Cultures From Neonatal Opossum *Monodelphis domestica*

**DOI:** 10.3389/fncel.2021.661492

**Published:** 2021-03-18

**Authors:** Antonela Petrović, Jelena Ban, Ivana Tomljanović, Marta Pongrac, Matea Ivaničić, Sanja Mikašinović, Miranda Mladinic

**Affiliations:** Laboratory for Molecular Neurobiology, Department of Biotechnology, University of Rijeka, Rijeka, Croatia

**Keywords:** opossums, cortex, primary neuron cell culture, radial glia cells, astrocytes, postnatal

## Abstract

Primary dissociated neuronal cultures have become a standard model for studying central nervous system (CNS) development. Such cultures are predominantly prepared from the hippocampus or cortex of rodents (mice and rats), while other mammals are less used. Here, we describe the establishment and extensive characterization of the primary dissociated neuronal cultures derived from the cortex of the gray South American short-tailed opossums, *Monodelphis domestica*. Opossums are unique in their ability to fully regenerate their CNS after an injury during their early postnatal development. Thus, we used cortex of postnatal day (P) 3–5 opossum to establish long-surviving and nearly pure neuronal cultures, as well as mixed cultures composed of radial glia cells (RGCs) in which their neurogenic and gliogenic potential was confirmed. Both types of cultures can survive for more than 1 month* in vitro*. We also prepared neuronal cultures from the P16–18 opossum cortex, which were composed of astrocytes and microglia, in addition to neurons. The long-surviving opossum primary dissociated neuronal cultures represent a novel mammalian *in vitro* platform particularly useful to study CNS development and regeneration.

## Introduction

A full understanding of the structure, function, and development of the mammalian central nervous system (CNS) is necessary to develop treatments that could allow successful regeneration of the adult injured or degenerated tissue. Mammalian developing CNS is challenging to study because of the extreme complexity of the dynamic events involved, including cell proliferation, migration, and differentiation. Moreover, it emerged that neurodegenerative diseases often originate during development, despite late onset (Schaefers and Teuchert-Noodt, 2016).

Dissociated primary neuronal cultures represent an excellent *in vitro* tool to study CNS development, as well as neuronal maturation and functional activity, at the single-cell level and at the network scale. These cultures allow us to gain mechanistic insights in a simplified but more controlled context, compared to *in vivo* conditions (Beaudoin et al., 2012; Humpel, 2015). The studies on primary neuronal cultures contributed to many fundamental discoveries regarding development such as neuronal polarization, neurite outgrowth, axon guidance (pathfinding), synaptogenesis and neuronal network formation, activity and maturation, recapitulating *in vitro* many aspects that occur *in vivo* (Cáceres et al., 2012; Kaech et al., 2012).

The most frequently used source for culturing primary neurons is the cortex or hippocampus of late embryonic or early postnatal rodents (i.e., rats and mice; Kaech and Banker, 2006; Beaudoin et al., 2012). Primary neurons derived from other mammalian species have been less frequently used. For instance, cortical primary neuronal cultures were recently obtained from developing porcine telencephalon (Aubid et al., 2019), sheep (Reddy et al., 2015), and marsupials such as opossum (Bartkowska et al., 2013), with the ability to maintain them *in vitro* for not more than 7, 5 or 2 days, respectively, suggesting their relatively limited period. Thus, in addition to rodents, there is a need to employ other mammalian species as a source for primary neuronal cultures, to identify the differences among mammals, and to avoid mistakes in translating the knowledge to humans (Bonfanti and Peretto, 2011; Rodemer et al., 2020).

In this work, we have established and extensively characterized long-term primary cortical dissociated cultures, prepared from the neonatal gray South American short-tailed opossums (*Monodelphis domestica*). Opossums are marsupials (Metatheria), which are mammalian infraclass that diverged from placental mammals (Eutheria) between 170 and 190 M years ago (Kumar and Hedges, 1998; Goodstadt et al., 2007; Dooley et al., 2013). Unlike the other marsupials, opossums such as *M. domestica* lack a pouch and the pups (usually from 1 to 12) are born after 14 days of gestation at a very immature state (Nicholls et al., 1990; Mladinic et al., 2009). Postnatal day 0 (P0) opossums are comparable to embryonic day 12 (E12) rat or E11.5 mice embryo, according to recent transcriptome analysis that matches previous estimates (Smith, 2001; Cardoso-Moreira et al., 2019). During the first two postnatal weeks, *M. domestica* neonates can fully regenerate the spinal cord after injury (Nicholls et al., 1994; Saunders et al., 1995, 1998; Varga et al., 1995; Nicholls and Saunders, 1996). This ability to fully regenerate the spinal cord after injury abruptly stops in opossums at the postnatal day 12 in the cervical part of the spinal cord and at the postnatal day 17 in less mature lumbar segments of the spinal cord (Wintzer et al., 2004; Mladinic et al., 2005, 2010). Moreover, fundamental brain development events such as the formation of hexalaminar cortex, cerebellum formation, and gliogenesis occur during the first month in opossums (Saunders et al., 1989; Molnár et al., 1998; Puzzolo and Mallamaci, 2010; Seelke et al., 2013; Tepper et al., 2020), offering the unique possibility to obtain developing (embryonic-like) CNS cells from postnatal animals, at a wide range of postnatal days.

Here, we describe the protocols to successfully develop and establish different *in vitro* primary dissociated cultures from developing cortex of postnatal opossums (*M. domestica*) of different ages, in which different CNS cell types predominate, including neurons, radial glia cells (RGCs), and astrocytes. We have chosen P3–5 and P16–18 opossums since those ages differ essentially in their capacity to regenerate spinal cord tissue after injury. Our protocols allow long-term survival of neuronal cell cultures (up to 1 month), as well as efficient maintenance and differentiation of RGCs into neurons* in vitro*. These *M. domestica*-derived primary CNS cultures are novel and robust *in vitro* platforms to use in a variety of studies, including development and regeneration.

## Materials and Methods

### Animals

In this work, South American gray short-tailed opossum (*Monodelphis domestica*) pups of both sexes at postnatal day (P)3–5 and 16–18 were used. The *M. domestica* colony was maintained at the animal house facility of the University of Trieste, following the guidelines of the Italian Animal Welfare Act, and their use was approved by the Local Veterinary Service, the Ethics Committee board, and the National Ministry of Health (Permit Number: 1FF80.N.9Q3), following the European Union guidelines for animal care (d.1.116/92; 86/609/C.E.). The animals are housed in standard laboratory cages in a temperature- and humidity-controlled environment (27–28°C; 50–60% humidity) with a 12/12 h light/dark cycle and *ad libitum* access to food and water. According to recently reported developmental transcriptome analysis (Cardoso-Moreira et al., 2019), P3–5 opossums correspond to E15.5–18.5 rat or E14–16 mice embryos, while P16–18 opossums are developmentally similar to neonatal (P1–P2) rat or mice. The body weight and size of animals through postnatal ages used in this study are shown in Table 1 and Supplementary Figure 1.

**Table 1:** Age, body weight and size of opossums used in this study.

Age	Body weight (g)	Body size (mm)
P3	0.14 ± 0.01	9.14 ± 0.48
P4	0.19 ± 0.02	11.50 ± 1.73
P5	0.22 ± 0.02	12.60 ± 0.42
P16	1.43 ± 0.04	31.20 ± 2.05
P17	1.63 ± 0.05	33.33 ± 1.15
P18	1.71 ± 0.11	33.00 ± 1.41

For each age 10–17 pups from at least three different litters were measured.

### Primary Dissociated Neuronal Cultures

The dissociation protocol was developed following the procedures previously described for rodents (Kaech and Banker, 2006; Beaudoin et al., 2012; Pozzi et al., 2017) and all efforts were made to minimize suffering and to reduce the number of animals used. Primary neurons were isolated from the cortex of neonatal opossums from two developmentally significant postnatal age groups: P3–5 and P16–18, respectively. Both left and right hemispheres from each animal were used while olfactory bulbs and remaining subcortical structures were removed. Dissection was performed in the ice-cold oxygenated (95% O_2_/5% CO_2_) dissection solution, consisted of 113 mM NaCl, 4.5 mM KCl, 1 mM MgCl_2_ × 6H_2_O, 25 mM NaHCO_3_, 1 mM NaH_2_PO_4_, 2 mM CaCl_2_ × 2H_2_O, 11 mM glucose and 0.5% w/v Penicillin/Streptomycin/Amphotericin B, pH 7.4 (all from Sigma–Aldrich, St. Louis, MO, USA). The meninges were removed, and the tissue was chopped into small pieces. Before enzymatic digestion, the cortices were washed three times with the sterile phosphate-buffered saline (PBS) containing 137 mM NaCl, 2.7 mM KCl, 10 mM Na_2_HPO_4_, 2 mM KH_2_PO_4_ (all from Sigma–Aldrich). Cortices were digested with prewarmed trypsin in PBS (Santa Cruz Biotechnology, SCBT, Dallas, TX, USA) by incubating at 32.5°C. The tissue from P3 to P5 pups was incubated in 0.5% w/v trypsin for 10 min, while that of P16–18 pups was incubated in 2.5% w/v trypsin for 15 min. After three washes with PBS, cells were dissociated using a 1 ml tip by pipetting up and down 15–20 times in a trituration solution containing 10 μg/ml DNAse I (Sigma–Aldrich), 1 mg/ml trypsin inhibitor (SCBT), and 1% w/v bovine serum albumin (BSA; Pan-Biotech GmbH, Aidenbach, Germany) in Hank’s Balanced Salt Solution (HBSS) solution, w/o Ca^2+^ and Mg^2+^ (Pan-Biotech). The supernatant containing dissociated cells was collected and layered on top of the 5% w/v BSA cushion in HBSS in the 5 ml tube to remove the cell debris. The trituration step was repeated two times for P3–5 and three times for P16–18 digested cortices. Cells were collected by centrifugation for 5 min at 100 *g* and then resuspended in a plating medium consisting of Dulbecco’s minimum essential medium (DMEM) with stable glutamine supplemented with 10% w/v fetal bovine serum (FBS) and 1% w/v Penicillin/Streptomycin (all from Pan-Biotech) and purified by preplating the cell suspension for 5 min on the plastic tissue culture dishes. Cells were counted using the hemocytometer and plated on glass coverslips (12 mm diameter) precoated with 50 μg/ml poly-L-ornithine and 2 μg/ml laminin (all from Sigma–Aldrich) at the density of 1 × 10^5^ cells per well in a 12-well plate or 5 × 10^4^ cells per well in a 24-well plate. The next day, 2/3 of the medium was changed with the neuronal medium containing Neurobasal medium supplemented with B27 (both from Thermo Fisher Scientific, Waltham, MA, USA), 1 mM L-glutamine, and 1% Penicillin/Streptomycin (both from Pan-Biotech). Subsequent media changes were done once per week changing only a half of the medium with the fresh neuronal medium. The cortical cultures were maintained in an incubator at 32°C, 5% CO_2_, and 95% relative humidity.

### Radial Glial Cell Cultures

Radial glial cells (RGCs) were prepared using the same protocol for dissection and dissociation as described for P3–5 neuronal cultures. Unlike neuronal cultures, RGCs were plated on poly-L-ornithine-coated dishes or coverslips without laminin at the density of 5 × 10^4^ cells per well in a 24-well plate. DMEM with stable glutamine supplemented with 10% w/v FBS and 1% w/v Penicillin/Streptomycin was used both for plating and culture. Cell passaging was done at 90%–100% confluence using 0.5% trypsin in PBS for 5 min at 32°C and subsequent replating on the poly-L-ornithine-coated glass coverslips in plating medium. To allow the formation of neurospheres, dissociated cells from the opossum cortex were cultured in a plating medium on a non-adherent tissue culture flask at the density of 6 × 10^5^ cells per 25 cm^2^ flask. The cells were transferred to a new tissue culture flask the next day to remove residual adherent cells such as fibroblasts. A quarter of the media was changed at DIV1 and DIV4. Neurospheres were grown until DIV7 (when they reached approximately 100 μm in diameter) and then transferred to poly-L-ornithine and laminin-coated glass coverslips in neuronal medium (Neurobasal medium supplemented with B27, see “Neuronal Cortical Cultures of P16-18 Opossums” section) to differentiate over the next 7 days.

### Immunofluorescence

Cells were fixed for 20 min at room temperature (RT, 20–22°C) with 4% paraformaldehyde (PFA, Sigma–Aldrich) containing 200 mM sucrose in PBS, pH 6.9. After fixation, cells were washed with PBS, saturated with 0.1 M glycine, permeabilized with 0.1% Triton X-100 (all from Sigma–Aldrich) in PBS, and lastly washed with PBS, each step lasting 5 min. Cells were blocked with 0.5% w/v BSA (Pan-Biotech) in PBS for 30 min. Incubation with the primary antibodies (Table 2) was done in a wet chamber for 1 h, followed by the washing steps in PBS and incubation with the secondary antibodies. For every primary antibody used, the protein sequence similarity between opossum and immunogen was compared using the Universal Protein Resource (UniProt^1^). The secondary antibodies were goat anti-mouse Alexa Fluor^®^ 555 (Thermo Fisher Scientific, Cat# A32732, RRID: AB_2633281, 1:400), goat anti-mouse Alexa Fluor^®^ 488 (Thermo Fisher Scientific, Cat# A32723, RRID: AB_2633275, 1:400), goat anti-rabbit Alexa Fluor^®^ 555 (Thermo Fisher Scientific, Cat# A32732, RRID: AB_2633281, 1:400), goat anti-rabbit Alexa Fluor^®^ 647 (Abcam, Cat# ab150083, RRID: AB_2714032, 1:300), goat anti-mouse immunoglobulin (Ig) G_1_ Alexa Fluor^®^ 488 (Thermo Fisher Scientific, Cat# A-21121, RRID: AB_2535764, 1:300) and goat anti-mouse IgG_2a_ Alexa Fluor^®^ 555 (Thermo Fisher Scientific, Cat# A-21137, RRID: AB_2535776, 1:300) and the incubation time was 30 min in the dark. For F-actin staining, Phalloidin-iFluor 488 (Abcam, Cambridge, UK) was incubated for 30 min together with the secondary antibody. Cell nuclei were stained with a 300 nM nuclear stain 4′,6-diamidino-2-phenylindole (DAPI, Thermo Fisher Scientific) diluted in PBS for 5 min. Finally, coverslips were washed with PBS and in dH_2_O. Coverslips were mounted on a glass slide using the mounting medium (Vectashield, Vector Laboratories, Burlingame, CA, USA) and sealed with nail polish. All the incubations were performed at RT. Proliferation assay was done using Click-iT^TM^ 5-ethynyl-2′-deoxyuridine (EdU) Cell Proliferation Kit, Alexa Fluor^TM^ 488 dye (Thermo Fisher Scientific). EdU was added to the cells according to manufacturer instruction immediately after the plating and incubated for 24 h. Cells were fixed at DIV1 and immunolabeled for 30 min at RT in Click-iT^®^ reaction cocktail. The cells were analyzed using an Olympus IX83 inverted fluorescent microscope (Olympus, Tokyo, Japan) equipped with differential interference contrast (DIC) and fluorescence optics (mirror units: U-FUNA: EX360–370, DM410, EM420–460, U-FBW: EX460–495, DM505, EM510IF, and U-FGW: EX530–550, DM570, EM575IF (Olympus) and Cy5 (EX620/60, DM660, EM700/75, Chroma, Irvine, CA, USA). Fluorescence images were acquired with Hamamatsu Orca R2 CCD camera (Hamamatsu Photonics, Hamamatsu, Japan) and CellSens software (Olympus, Japan). 10× 0.3 numerical aperture (NA) air, 20× 0.5 NA air, and 40× 1.4 NA oil immersion objectives were used. For each image 15–30 slices were acquired with slice spacing of 1 μm (10× and 20× objectives) and 0.3–0.5 μm (40× objective). For each image, a maximum intensity projection was used. CellSens and ImageJ by W. Rasband (developed at the U.S. National Institutes of Health^2^) were used for image processing and analysis. For GC, soma, and nuclei surface area analysis, the contour of each region of interest (ROI) was manually traced, and the surface was measured using ImageJ/Fiji.

**Table 2:** Primary antibodies used in this work.

Antibody	Host and isotype	Dilution	Immunogen	Producer Cat # RRID	UniProt Identity
Glutamic acid decarboxylase 65 (GAD65)	Mouse monoclonal IgG1	1:200	Rat brain GAD, purified using Protein A chromatography	Sigma–Aldrich SAB4200232
AB_10762670	86.6%
Glial fibrillary acidic protein (GFAP)	Mouse monoclonal IgG_1_	1:200	GFAP from porcine spinal cord	Sigma–Aldrich G3893
AB_477010	84.9%
Ionized calcium-binding adaptor molecule 1 (Iba1)	Rabbit polyclonal	1:100	Synthetic peptide corresponding to Human Iba1 (C terminal)	Abcam ab15580 AB_10862652	63.4%
Microtubule-associated protein 2 (MAP2)	Mouse monoclonal IgG_1_	1:200	Bovine MAP2	Sigma–Aldrich M1406 AB_477171	82.8%
Neuronal nuclei (NeuN)	Rabbit monoclonal	1:200	Synthetic peptide within Human NeuN aa 1–100 (Cysteine residue)	Abcam ab177487 AB_2532109	84%
Paired box gene 2 (PAX2)	Rabbit monoclonal IgG	1:50	Synthetic peptide within Human Pax2 aa 1–100	Abcam ab79389 AB_1603338	94.5%
Synapsin 1	Rabbit polyclonal	1:100	Synapsin I (a mixture of Ia and Ib) purified from bovine brain	Millipore AB1543 AB_2200400	72.3%
β-Tubulin III (TUJ1)	Mouse monoclonal IgG_2a_	1:200	Microtubules derived from rat brain	Biolegend 801201 AB_2313773	99.8%
Vesicular Glutamate Transporter 2 (vGLUT2)	Guinea pig polyclonal	1:200	Synthetic peptide from rat VGLUT2 protein	Chemicon AB5907 AB_2301731	96.4%

### Statistical Analysis

All results have been obtained from at least three independent experiments and are presented as a bar graph with mean ± SEM. Statistical analysis was performed using GraphPad Prism 8.4 (GraphPad Software Inc., La Jolla, CA, USA). To test the normality of data, depending on the number of values tested, either D’Agostino–Pearson or Shapiro–Wilk normality test was used. Brown–Forsythe test was used to test the equality of variances. One-way ANOVA was used to compare three or more data groups when data followed Gaussian distribution. Following the one-way ANOVA, the Holm–Šídák test was performed for multiple comparisons between data groups. Data groups with different variances that fail a normality test were compared using the Kruskal–Wallis test. Following the Kruskal–Wallis test, Dunn’s multiple comparisons test was performed for multiple comparisons between data groups. *T*-test was used when comparing two normally distributed data groups with equal variances, and in case of unequal variances, Welch’s *t*-test was used. The accepted level of significance was *p* < 0.05. *p* < 0.001 very significant ***, 0.001–0.01 very significant **, 0.01–0.05 significant *, ≥0.05 not significant.

## Results

### Establishment of Neuronal Cultures From P3 to P5 Opossum Cortex

As described in “Materials and Methods” section in detail, the dissection protocol was adapted from the standard and well-established protocols for rodent embryonic or postnatal neuronal cultures from cortex or hippocampus (Kaech and Banker, 2006; Beaudoin et al., 2012), with some modifications. In particular, to preserve cell viability during the dissection procedure, an ice-cold oxygenated dissection medium was used. We introduced two-step enzymatic digestion, bovine serum albumin (BSA) cushion, and preplating steps (see “Materials and Methods” section). Importantly, since opossums have lower body temperature than most placental mammals and slightly lower than other marsupials (Harder et al., 1996), their primary cultures have been maintained at 32°C, as previously described for organotypic cultures derived from developing opossum cortex (Puzzolo and Mallamaci, 2010).

#### *M. domestica*-Derived Neuronal Growth Cones (GCs)

**Figure 1:**
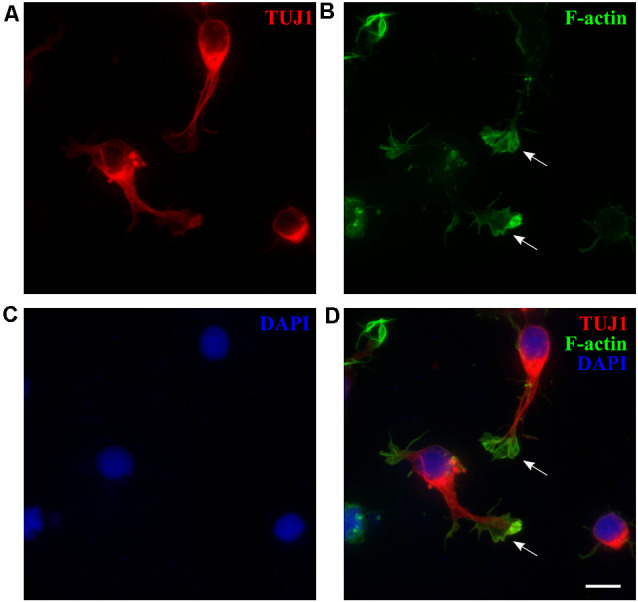
*M. domestica*-derived neurons and neuronal growth cones (GCs) at DIV1. Primary neuronal cultures prepared from P5 opossums were fixed 24 h after plating and stained for **(A)** β-tubulin III (TUJ1, red), **(B)** filamentous (F)-actin (green), **(C)** nuclear stain 4′,6-diamidino-2-phenylindole (DAPI; blue) and **(D)** merged. Arrows indicate GCs identified with F-actin immunostaining. Scale bar, 10 μm.

Cortical dissociated neurons from P3 to P5 opossums attached and survived well after one day *in vitro* (DIV1) and were positive for β-tubulin III marker (TUJ1, Figure 1A), specific for early postmitotic neurons (Menezes and Luskin, 1994; von Bohlen Und Halbach, 2007). Growth cones (GCs), highly dynamic actin-rich structures that play an essential role in axon guidance (Dent et al., 2011; Vitriol and Zheng, 2012), were formed at the tips of the growing neurites, as shown in Figure 1B. We analyzed the size of *M. domestica*-derived GCs using phalloidin (F-actin) fluorescence stain (Figure 1B). The GCs had variable sizes with an average value of 39.50 ± 3.09 μm^2^ (*n* = 40). To the best of our knowledge, these are the first experimental observations showing that neuronal GCs derived from the opossum cortex can be efficiently obtained *in vitro*, with a size comparable to rat hippocampal GCs (Pozzi et al., 2017).

#### Neuronal Outgrowth and Expression of Neuronal Markers

Next, we explored the expression of different neuronal markers in opossum cortical primary cultures. For instance, NeuN, neuron-specific nuclear protein widely expressed in vertebrates (Mullen et al., 1992) has been previously detected on cortical sections of developing opossums by immunohistochemistry (Seelke et al., 2013; Bartkowska et al., 2014), while microtubule-associated protein (MAP)2 is a conventional somatodendritic neuronal marker (Binder et al., 1986; Menezes and Luskin, 1994; Dehmelt and Halpain, 2005).

**Figure 2:**
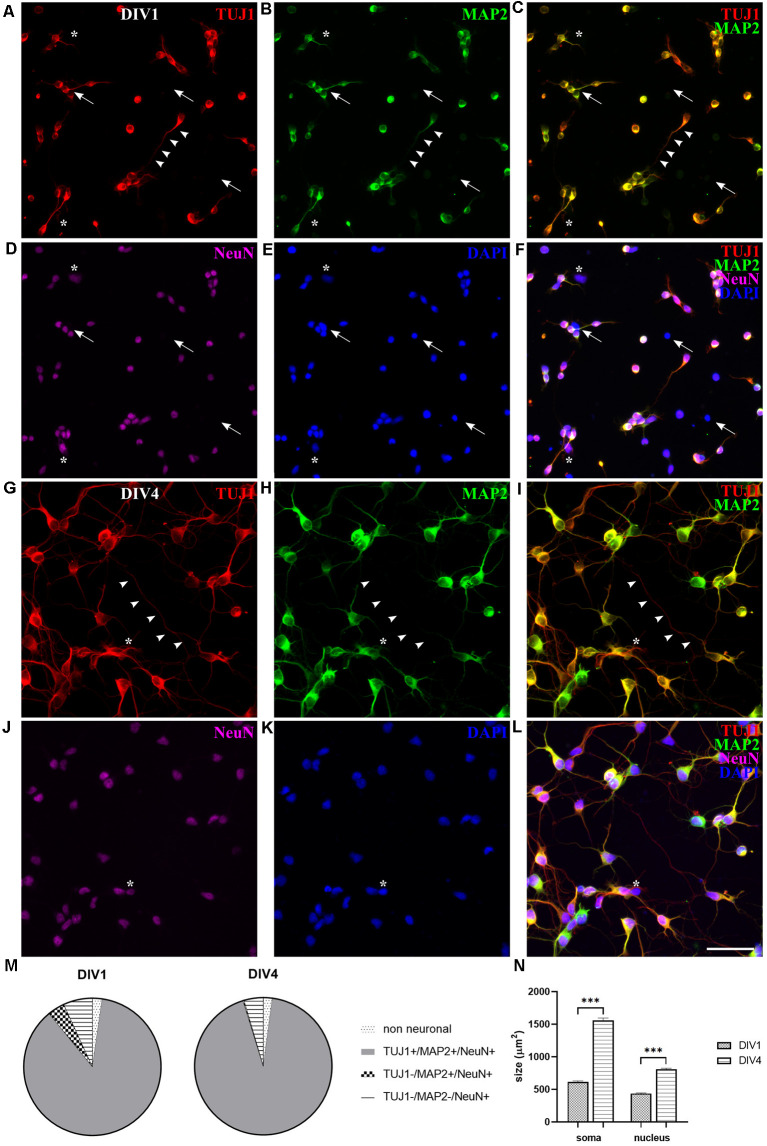
Expression of neuronal markers in cortical cultures derived from P5 opossum. Cells were fixed at DIV1 and stained for **(A)** TUJ1 (red), **(B)** MAP2 (green), **(D)** NeuN (magenta), and **(E)** DAPI nuclear stain (blue). **(C)** Merged image of TUJ1 and MAP2. **(F)** Merged image of all markers. **(G–L)** Same as **(A–F)** but for DIV4 cultures. Asterisks indicate TUJ1^−^/MAP2^−^/NeuN^+^ and arrows indicate non-neuronal (TUJ1^−^/MAP2^−^/NeuN^−^) cells. Arrowheads indicate TUJ1^+^/MAP2^−^ axon. **(M)** Percentage of expression for TUJ1, MAP2, and NeuN at DIV1 and DIV4, respectively. **(N)** Neuronal cell bodies (soma) and neuronal nuclei size increased from DIV1 to DIV4. Unpaired t-test with Welch’s correction. DIV1 soma vs. DIV4 soma *p* < 0.001***, DIV1 nucleus vs. DIV4 nucleus *p* < 0.001***. Scale bar, 50 μm.

At DIV1, NeuN, MAP2, and TUJ1 neuronal markers were already detected in cultures of dissociated P3–5 opossums’ neurons (Figure 2). The majority of the cells (86.83 ± 0.98%, *n* = 367) were triple positive for TUJ1, MAP2, and NeuN (TUJ1^+^/MAP2^+^/NeuN^+^, Figure 2F). The lower portion of neurons (6.86 ± 0.76%, *n* = 367) was expressing only NeuN (TUJ1^−^/MAP2^−^/NeuN^+^, Figures 2A–F; asterisks) and TUJ1-negative neurons were also found (TUJ1^−^/MAP2^+^/NeuN^+^, 4.02 ± 0.85%, *n* = 367). Finally, triple-negative non-neuronal cells (TUJ1^−^/MAP2^−^/NeuN^−^) were 2.28 ± 0.35% of all cells (*n* = 367, Figures 2A–F; arrows). These results suggest that at DIV1 the expression of NeuN slightly precedes MAP2 and TUJ1, with the vast majority of neurons expressing all three neuronal markers (Figure 2M).

At DIV4 virtually all (>99%) neurons present in culture coexpressed TUJ1 and MAP2, while only a minor portion was made up by TUJ1-negative neurons, expressing MAP2 and NeuN markers (TUJ1^−^/MAP2^+^/NeuN^+^, 0.26 ± 0.54%, *n* = 442). Neurons positive only to NeuN marker (TUJ1^−^/MAP2^−^/NeuN^+^ cells) were reduced to 4.53 ± 0.55% (*n* = 442). However, at DIV4 the total number of neurons (TUJ1, MAP2, and NeuN triple-positive cells) exceeded hugely the number of non-neuronal cells, which was further reduced to around 2% (TUJ1^−^/MAP2^−^/NeuN^−^ cells, 2.11 ± 0.48%, *n* = 442), indicating the progression of neuronal differentiation in culture (Figure 2M).

Since TUJ1 and MAP2 are also cytoskeletal markers, we used them to follow *in vitro* neurite outgrowth in opossum primary cultures. Similar to rodents (Dotti et al., 1988; Cáceres et al., 2012), at DIV1 in *M. domestica*-derived cortical neuronal cultures it was not possible to distinguish axons from dendrites as TUJ1 and MAP2 were coexpressed in all neurites (Figures 2A–C). In contrast, at DIV4, axons were identified as TUJ1^+^/MAP2^−^ neurites (Figures 2G–L, arrowheads). At DIV4, we counted on average 4.70 ± 0.13 neurites per neuron (the total number of TUJ1-positive neurites emerging from soma; 170 neurons analyzed). This result is similar to rat hippocampal neuronal cultures cultured in Neurobasal/B27-based medium (Pozzi et al., 2017), where also the enhanced branching was observed in the first 4 days *in vitro*.

Moreover, the 2.5-fold increase of neuronal cell bodies was also observed at DIV4: at DIV1 average soma surface area was 198.92 ± 4.62 μm^2^ (*n* = 83) while at DIV4 it reached 503.10 ± 11.15 μm^2^ (*n* = 95). Nuclei surface increased 1.8-fold: from 140.93 ± 3.01 μm^2^ (*n* = 83) at DIV1–261.03 ± 4.77 μm^2^ (*n* = 95) at DIV4. This analysis was done using MAP2 and DAPI staining for soma and nuclei, respectively (see “Materials and Methods” section). Our *in vitro* observations correlate with the increase in average neuron size observed *in vivo*, during the first postnatal week in the rat brain (Bandeira et al., 2009).

#### *In vitro* Neuronal Maturation and Synapse Formation

**Figure 3:**
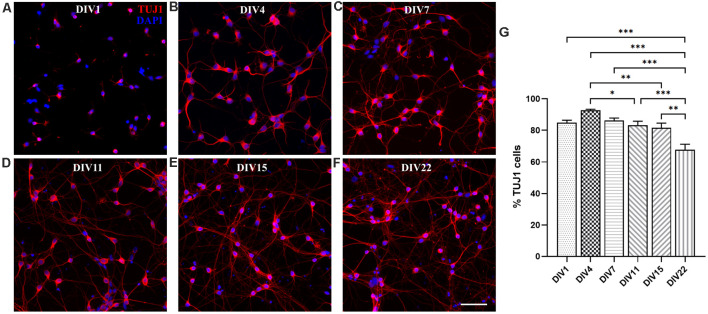
Primary neuronal cultures derived from the P3 to P5 cortex of *M. domestica*. Neurons were stained for β-tubulin III (TUJ1, red) and nuclei were stained with DAPI (blue). The cells were fixed and stained at **(A)** DIV1, **(B)** DIV4, **(C)** DIV7, **(D)** DIV11, **(E)** DIV15, and **(F)** DIV22, respectively. For better visualization of neurites, the grayscale images are available in Supplementary Figure 2. **(G)** Histogram showing percentage of neurons at the times indicated in panels **(A–F)**. One-way ANOVA followed by Holm–Šídák multiple comparisons test. DIV4 vs. DIV11 *p* = 0.0395*, DIV4 vs. DIV15 *p* = 0.00644**, DIV1 vs. DIV22 *p* < 0.001***, DIV4 vs. DIV22 *p* < 0.001***, DIV7 vs. DIV22 *p* < 0.001***, DIV15 vs. DIV22 *p* = 0.0013**. Scale bar, 50 μm.

We analyzed *in vitro* maturation and long-term survival of opossum cortical cultures, as well as progressive neuronal network formation. Figure 3 shows neuronal network formation during the 3 weeks *in vitro*. At DIV1 (Figures 2A–F, 3A, Supplementary Figure 2), following dissociation and plating, neurons started to regrow their processes and established first connections with the neighboring cells. We counted the percentage of TUJ1-positive neurons among total cells at DIV1 (84.82 ± 4.87%, *n* = 663) and for all time points considered (Figure 3G). TUJ1 was the neuronal marker of choice because it stains both dendrites and axons (for better visualization of neurites, the grayscale images are available in Supplementary Figure 2). After 4 days in culture in serum-free Neurobasal/B27-based medium, the highest percentage of neurons (92.66 ± 2.02%, *n* = 603) was reached (Figures 3B,G), resulting in nearly pure neuronal cultures. At DIV7, 86.28 ± 4.25% (*n* = 980) of cells were TUJ1^+^ neurons (Figure 3C) and virtually all neurons have established connections with each other. At DIV11 and DIV15, the proportion of neurons remained above 80% (DIV11: 83.28 ± 2.50, *n* = 637 and DIV15: 81.53 ± 2.99%, *n* = 560, Figures 3D,E), while at DIV22 it decreased to 67.68 ± 3.45% (*n* = 633, Figure 3F). Our results have shown the striking and efficient neuronal survival during the first 2 weeks *in vitro*, results very similar to those obtained with E18 rat cortical neurons (Kim et al., 2007; Cullen et al., 2010).

We were able to maintain opossum cortical cultures up to 1 month *in vitro* (Supplementary Figure 3), although the cell survival decreased significantly after 3 weeks. Starting from DIV15, the formation of cell clusters was observed, and this became progressively more evident in the following days (Supplementary Figure 3). These clusters were organized in spherical aggregates of neuronal cell bodies from which neurites grew with parallel orientation. To form the aggregates, the cells necessarily migrated *in vitro*, since the tissue was initially dissociated and plated as single-cell suspension (see Figure 3A). Similar structures were previously described in long-term (DIV67) rat hippocampal cultures (Todd et al., 2013). We confirmed the expression of TUJ1 as well as MAP2 throughout the observation period, including DIV30, using several different antibodies for each marker (Supplementary Figures 4, 5).

**Figure 4:**
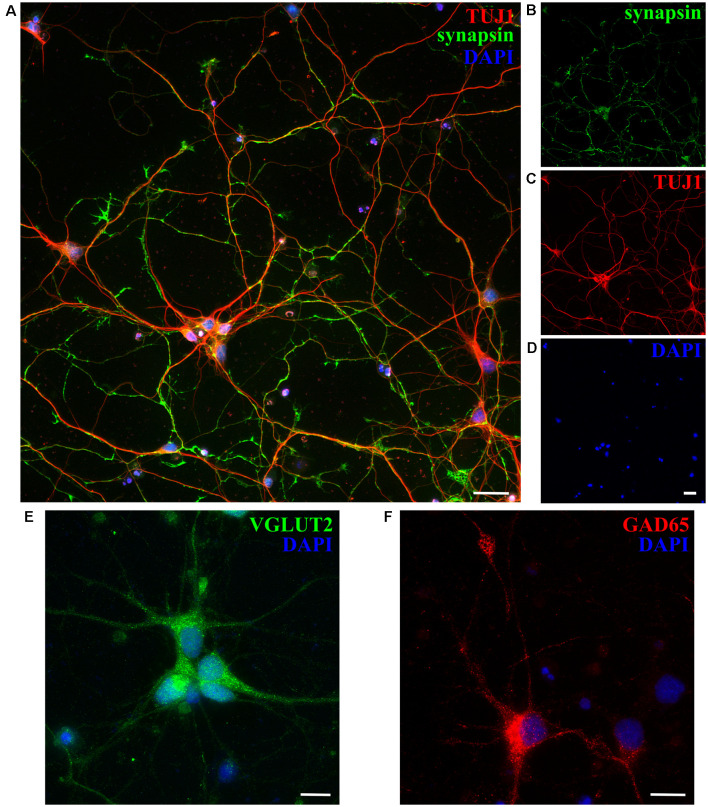
Expression of synaptic and neuronal subtype markers at DIV15 of cortical cultures obtained from P5 opossums. **(A)** Merged image of staining for **(B)** synapsin (green; **C**) β-tubulin III (TUJ1, red) and **(D)** DAPI nuclear stain (blue).** (E)** Excitatory VGLUT2-positive (green), **(F)** GABAergic inhibitory GAD65-positive (red) neurons, and DAPI-positive nuclei (blue), respectively. Scale bar, 25 μm.

Next, we checked the expression of synaptic markers such as synapsin in opossum neuronal cultures, using previously described protocols (Cullen et al., 2010; Todd et al., 2013; Petrovic et al., 2019) and confirming the formation of synaptic connections and the formation of *in vitro* neuronal networks at DIV15 (Figures 4A–D). Moreover, both excitatory and inhibitory neuronal subtypes were found, using as markers vesicular glutamate transporter 2 (VGLUT2) and glutamic acid decarboxylase (GAD65), respectively (Figures 4E,F).

### Neuronal Cortical Cultures of P16–18 Opossums

In addition to neuronal cultures derived from P3 to P5 neonates, we also established primary cortical cultures from P16 to P18 opossums. At this age, pups are around 3 cm long and their body weight is around 1.5 g (Table 1, Supplementary Figure 1). They are developmentally equivalent to P0–3 rats or mice (Cardoso-Moreira et al., 2019), whose primary cortical and hippocampal cultures are well characterized (Beaudoin et al., 2012). The cortex of older animals is in general more difficult to dissociate and therefore few additional modifications regarding the protocol for P3–5 opossums were introduced: trypsin concentration and incubation time were increased with additional pipetting during dissociation.

**Figure 5:**
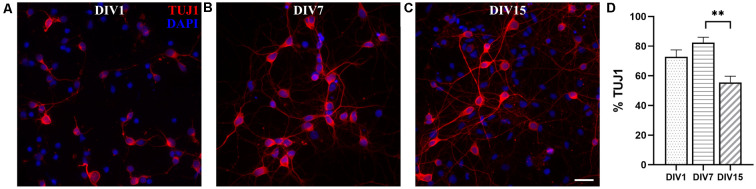
Primary dissociated cortical cultures derived from P16 to P18 opossums. **(A)** DIV1, **(B)** DIV7, and **(C)** DIV15 neuronal cultures stained for β-tubulin III (red). Nuclei were stained with DAPI (blue). **(D)** Histogram showing percentage of neurons at the time points indicated in panels **(A–C)**. Kruskal–Wallis test followed by Dunn’s multiple comparisons test. DIV7 vs. DIV15 *p* = 0.0071**. Scale bar, 25 μm.

Neuronal cultures derived from P16 to P18 opossums followed similar dynamics of network formation to those observed in P3 to P5 cortical cultures, during the first 2 weeks *in vitro* (Figure 5A). At DIV1, 72.80 ± 4.67% cells were TUJ^+^ (*n* = 2,070, Figures 5A,D) and their proportion increased to 82.37 ± 3.67% (*n* = 704, Figures 5B,D) at DIV7. At DIV15, the percentage of neurons was significantly reduced to 55.59 ± 4.15% (*n* = 1,514, Figures 5C,D). Although it was possible to keep neuronal cultures derived from P16 to P18 opossum *in vitro* up to 1 month (Supplementary Figure 5), their lower long-term survival was observed, compared to cultures derived from P3 to P5 animals. The lower percentage of neurons present in cultures derived from older animals (P16–18) reflects the situation *in vivo*, as well as it is probably related to the higher susceptibility of P16–18 neurons to the dissociation process, as shown in mice hippocampal or cortical primary cultures (Beaudoin et al., 2012).

### Non-neuronal Cells

Gliogenesis occurs at late embryonic and postnatal age in rodents (Kaech and Banker, 2006; Malatesta et al., 2008), while in opossums it presumably starts around P18 when the switch from radial glia to astrocytes morphology is first observed (Puzzolo and Mallamaci, 2010). Thus, we analyzed the content of non-neuronal cells in opossum cortical cultures, derived from both P3–5 and P16–18 opossums, in which the percentage of neurons is much smaller.

**Figure 6:**
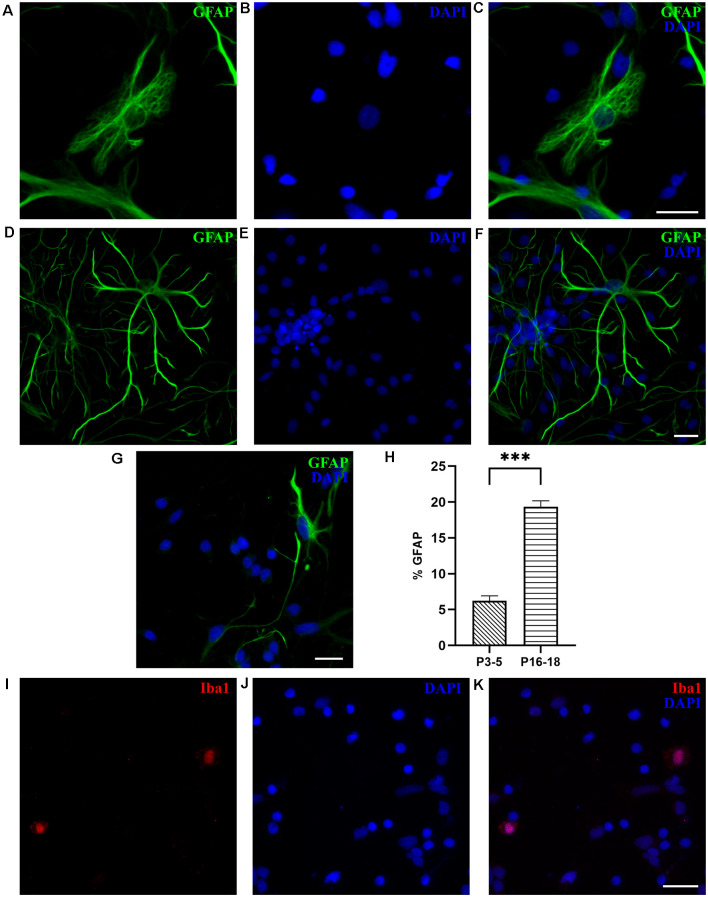
Glial cells in cortical cultures prepared from P16 to P18 opossums. **(A)** Astrocytes at DIV7 primary cultures derived from the P17 cortex with protoplasmic-like morphology were stained for glial fibrillary acidic protein (GFAP, green).** (B)** Cell nuclei were stained with DAPI (blue). **(C)** Merged images of panels **(A,B)**. **(D–F)** Same as panels **(A–C)** showing astrocytes with stellate morphology. **(G)** DIV7 primary astrocytes derived from the P4 cortex and stained for GFAP (green) and DAPI (blue). **(H)** Histogram showing percentage of GFAP-positive cells among total cells, stained with DAPI, from primary cultures derived from different postnatal ages (P3–5 and P16–18, respectively). Unpaired *t*-test. P3–5 vs. P16–18 *p* < 0.001***. **(I)** Microglia in cortical cultures derived from P18 opossum, fixed at DIV1 and immunostained for microglial marker Iba1 (red). **(J)** DAPI nuclear stain and **(K)** merged image. Scale bar, 25 μm.

To identify astrocytes, we used glial fibrillary acidic protein (GFAP), the most commonly used marker of astrocytes, which in *M. domestica* was previously used on tissue slices (Puzzolo and Mallamaci, 2010). As shown in Figure 6, at DIV7, GFAP-positive cells were present, as both protoplasmic-like (Figures 6A–C) and stellate astrocytes (Figures 6D–F), as previously observed in rodent cultures (Verstraelen et al., 2014; Wolfes et al., 2017). In contrast to P16–18 cultures, primary astrocytes prepared from the P3–5 cortex showed less elaborated, thinner, and elongated shapes, indicating a lower degree of maturity (Figure 6G). The GFAP-positive cells were less abundant in cultures derived from P3 to P5 opossums, presenting only 5.71 ± 0.53% of total cells (*n* = 1,948, Figure 6H), vs. 19.35 ± 0.83% (*n* = 1,085) of GFAP^+^ cells in cultures derived from P16 to P18 cortex. In addition to GFAP, we confirmed the expression of intermediate filament protein vimentin (Eliasson et al., 1999; de Pablo et al., 2019) in developing astrocytes derived from both P3–5 and P16–18 opossums (Supplementary Figure 6). *M. domestica*-derived astrocytes’ morphology is strikingly similar to those derived from rodents (Verstraelen et al., 2014; Ulloa Severino et al., 2016; Pozzi et al., 2017; Wolfes et al., 2017), offering the new source of mammalian astrocytes for further *in vitro* investigations.

In addition to astrocytes, Iba1-positive microglial cells were found at DIV1 in cultures derived from the P18 cortex, accounting for 2.15 ± 0.71% of cells (*n* = 531, Figures 6G–I). We were not able to identify any microglia in cultures derived from P3 to P5 cortex, nor oligodendrocytes, neither in P3–5 or P16–18-derived cultures, likely because the oligodendrogenesis in opossums occurs around P40 (Puzzolo and Mallamaci, 2010).

### Radial Glia Cells

RGCs represent the main population of neural progenitors in the developing cortex (Malatesta et al., 2008). Opossum neonates are equivalent to rodent embryos and most of the cortical neurogenesis occurs after the birth with first cortical layering observed around P4 (Saunders et al., 1989; Puzzolo and Mallamaci, 2010). Therefore, in the primary cultures derived from postnatal opossum brains, we expected the presence of proliferative progenitor/radial glial cells. To evaluate the proliferative state of P3–5-derived cultures, we performed 5-ethynyl-2′-deoxyuridine (EdU)-based proliferation assay at DIV1 (see “Materials and Methods” section) and obtained 18.15 ± 5.36% EdU^+^ cells (*n* = 575, Supplementary Figure 7). TUJ1 and EdU showed exclusive staining confirming that TUJ1 is expressed only in post-mitotic neurons while double-positive GFAP^+^/EdU^+^ cells confirmed the proliferative state of RGCs (Supplementary Figure 8).

**Figure 7:**
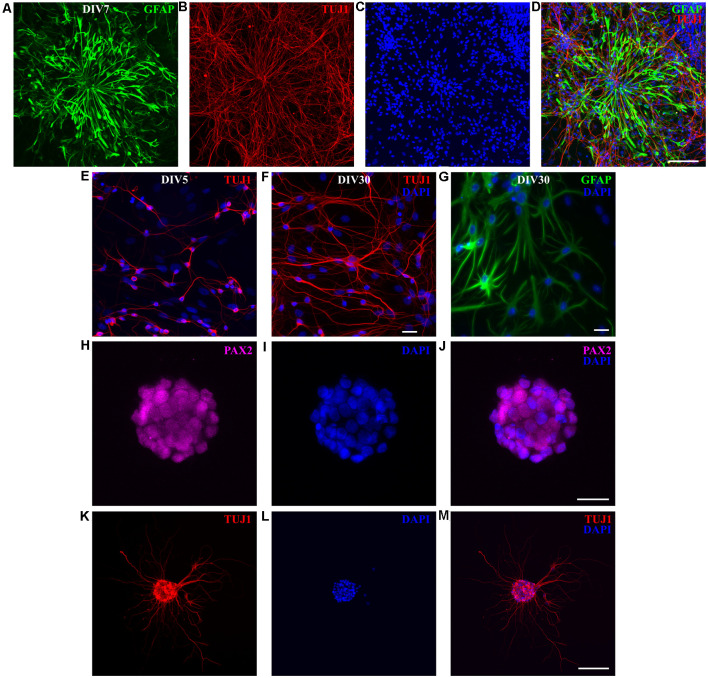
P3 opossum-derived radial glial cells (RGCs) and neurospheres. **(A)** DIV7 primary cultures derived from P3 cortex stained for glial fibrillary acidic protein (GFAP, green), **(B)** TUJ1 (red), and **(C)** nuclear stain DAPI (blue). **(D)** Merged image. Scale bar, 100 μm. Neuronal cultures **(E)** 5 and **(F)** 30 days after replating, stained for TUJ1 (red) and DAPI (blue). **(G)** Same as **(F)** but for GFAP (green) and DAPI (blue). **(H–M)** Immunocytochemical characterization and development of neurospheres. **(H–J)** DIV7 neurospheres in proliferation-promoting medium, stained for PAX2 (magenta), DAPI (blue), and merge, respectively. Scale bar, 25 μm. **(K)** DIV7 neurospheres attached on poly-L-ornithine- and laminin-coated glass coverslips and cultured in the serum-free neuronal medium for additional 7 days extend long processes and express TUJ1 (red). **(L)** Nuclear stain (DAPI, blue) and **(M)** merge. Scale bar, 100 μm.

To maintain proliferative cell state, instead of using serum-free Neurobasal/B27 medium (Brewer et al., 1993), used to obtain almost pure neuronal cultures (see Figures 2, 3), we kept the cultures in FBS-based medium, for 1 week. At DIV7, RGC-like GFAP-positive cells were observed (Figures 7A–D), resembling RGCs because of their bipolar and elongated morphology, with processes that extended for several hundreds of micrometers. As in previously described neuronal cultures, GFAP and TUJ1 had exclusive staining. The presence of postmitotic TUJ1-positive neurons showed that effective neuronal differentiation occurred despite the presence of FBS in the culture medium (Figure 7B). Overall cell density increased with time (compare DIV7 cultures shown in Figure 3C with Figures 7A–D). We, therefore, assumed that new RGC and new neurons were generated *in vitro*, in addition to preexisting neurons (plated at “DIV0”).

To verify the *in vitro* neurogenic potential of RGCs, we replated the cultures maintained for 1 month *in vitro*. Cells were trypsinized and replated on poly-L-ornithine-coated glass coverslips in a plating medium (see “Materials and Methods” section). Five days after replating, TUJ1-positive neurons were found (Figure 7E). These cultures were able to survive for an additional 1 month and they were composed of both neurons (Figure 7F) and astrocytes (Figure 7G). We have successfully replated these cultures for up to four passages (unpublished observations). These data showed that opossum-derived RGCs have both neurogenic and gliogenic *in vitro* potential. Moreover, the fact that cells can be maintained and expanded in culture for more than two months significantly reduces the number of sacrificed animals.

Finally, to additionally assess the *in vitro* neurogenic potential of opossum-derived RGCs, we performed a neurosphere assay (Ferrari et al., 2010; Pastrana et al., 2011) by culturing dissociated cortical cells in suspension, using non-adherent tissue culture surfaces in proliferative FBS-based medium. Dissociated cortical cells formed floating spherical aggregates of approximately 100 μm in diameter. These neurospheres were positive for paired box gene 2 (PAX2, Figures 7H–J), an early marker of neural progenitors during CNS development (Terzić et al., 1998; Vinci et al., 2016). When plated on the adhesive substrate (poly-L-ornithine- and laminin-coated coverslips) in differentiation promoting, serum-free medium, neurospheres extended long processes and expressed TUJ1 (Figures 7K–M). These results confirmed that RGCs derived from opossums have neurogenic properties and opened the perspective to further investigations, including those aiming to develop successful cell replacement therapies after CNS injury or degeneration.

## Discussion

In this study, we have shown that long-term primary neuronal cultures can be successfully obtained from neonatal opossums (*M. domestica*) and that with the use of the animals of different postnatal age, as well of different procedures and media, the enrichment of different CNS cell types, such as neurons or RGCc, can be obtained. Moreover, we have shown that both neuronal and astrocyte differentiation, the formation of GCs, synapses, and neurospheres as well as RGCs proliferation occurs *in vitro*, which makes opossum cortical cultures a favorable platform for investigating CNS development and regeneration.

### *M. domestica* as a Favorable Source of CNS Cells

There are several advantages in using opossums as the CNS cell source (Saunders et al., 1989; Nicholls et al., 1990; Seelke et al., 2013). First, the relatively short gestation (1 week shorter than rodents) allows easier and faster breeding. Second, they lack pouch making pups easily accessible. Third, opossum neonates are born very immature, offering embryonic-like tissue in postnatal ages. The use of postnatal animals reduces the number of animals used and their suffering, contributing to the 3Rs (reduce, refine, replace; Russell and Burch, 1960; Beaudoin et al., 2012; Humpel, 2015). Next, having extended postnatal CNS development, with neuro- and gliogenesis occurring almost completely postnatally, opossums offer a wide time-window for studying CNS development. Most of all, opossums are unique among mammals for being able to postnatally regenerate their spinal cord after injury (Saunders et al., 1995; Nicholls and Saunders, 1996). Namely, the injured spinal cord tissue of the neonatal opossums can be completely functionally regenerated until the P12 in the upper cervical part, and until the P17 in the lower, less mature thoracic-lumbar segments of the spinal cord. This capacity has been studied *in vivo* (Saunders et al., 1995; Molnár et al., 1998) or *in vitro* using intact spinal cord in culture (Treherne et al., 1992; Woodward et al., 1993; Varga et al., 1996). However, both approaches do not allow easy molecular or pharmacological manipulations, like cell cultures, which are easier to maintain, manipulate and analyze.

Most of the existing *in vitro* models have been obtained from rodents and there is a need to employ more mammalian species to identify the differences among mammals and to avoid mistakes in extrapolating this knowledge to humans (Bonfanti and Peretto, 2011; Rodemer et al., 2020). Therefore, opossums represent an alternative mammalian model for development and regeneration as well as comparative evolutionary studies.

According to recently reported developmental transcriptome analysis (Cardoso-Moreira et al., 2019), P3–5 opossums correspond to E15.5–18.5 rat or E14–16 mice embryos, while P16–18 opossums are developmentally similar to neonatal (P1-P2) rat or mice. Primary neuronal cultures from embryonic rat or mice are mostly composed of neurons (Brewer et al., 1993; Kaech and Banker, 2006), while those prepared from early postnatal rodents contain a higher proportion (between 10% and 20%) of glial cells (Beaudoin et al., 2012; Todd et al., 2013; Ulloa Severino et al., 2016). Opossum-derived cultures described in this work, reflect these developmental differences very well, offering the possibility of interspecies comparisons.

### Almost Pure Neuronal Cultures Obtained From the Cortex of P3–5 Opossums

Using the P3–5 opossums’ cortex, almost pure (>93%) neuronal cultures have been obtained at DIV4, resembling E16–18 mice or rat cortical cultures with >90% neurons (Kaech and Banker, 2006). Starting from DIV1, the cells expressing different neuronal markers were detected, with the majority of the cells (>86%) being triple positive for TUJ1, MAP2, and NeuN markers. A lower portion of neurons (around 7%) was expressing only NeuN marker, while non-neuronal cells (triple-negative for TUJ1, MAP2, and NeuN) represented only around 2% of all cells. At DIV4 the percentage of neurons further increased, with the number of non-neuronal cells stable around 2%. Neurons positive only to NeuN marker (and negative to TUJ1 and MAP2 markers) were further reduced to <5%, indicating the progression of neuronal differentiation *in vitro*. In addition to immunohistochemistry performed on cortical slices of developing or adult opossums (Bartkowska et al., 2013, 2014; Seelke et al., 2013, 2014), here we show for the first time MAP2 and NeuN expression in primary dissociated cortical cultures of *M. domestica*.

*In vitro* axonal formation was detected at DIV4, where on average 4.70 ± 0.13 neurites per neuron were counted. These results are similar to rat hippocampal neuronal cultures (Pozzi et al., 2017), where also the enhanced branching was observed in the first 4 days *in vitro*. Moreover, we also detected the 2.5-fold increase of neuronal cell bodies at DIV4, with the average soma surface area for neurons around 200 μm^2^. These observations correlate well with the increase in average neuron size observed *in vivo*, during the first postnatal week in the rat brain (Bandeira et al., 2009).

### *M. domestica* Neuronal Growth Cones *In vitro*

In dissociated cortical neurons derived from P3 to P5 opossums numerous GCs, key elements involved in axon guidance (Dent and Gertler, 2003; Dent et al., 2011; Vitriol and Zheng, 2012), were formed at the tips of the growing neurites at DIV1.

The size of the GCs can vary significantly between different species. Invertebrates such as *Aplysia californica* have “giant” GCs with a surface area of around 1,000 μm^2^, while vertebrate GCs are 10× smaller (Ren and Suter, 2016). We found that *M. domestica* GCs had variable sizes with an average value of 39.50 ± 3.09 μm^2^, the size comparable with vertebrate GCs (<100 μm^2^; Ren and Suter, 2016). In particular, the rat hippocampal GCs, fixed and stained at the same time point and cultured in a similar, FBS-based medium, measure around 36 μm^2^ (Pozzi et al., 2017). To the best of our knowledge, here we report for the first time the protocol for efficient generation of *in vitro* neuronal GCs derived from opossum cortex, with the size comparable to rat hippocampal GCs, allowing investigations of axon guidance using a novel mammalian *in vitro* model. Further investigations could explore the expression of receptors for guidance molecules, growth factors, or neurotrophins, as well as cytoskeletal dynamics in opossums as additional information for mammalian CNS development and regeneration studies.

### Primary Neuronal Cultures Derived From P16 to P18 Opossum Cortex

We successfully established primary cultures from the P16–18 opossum cortex. Unlike cortical cultures prepared from P3 to P5 opossums, P16–18 cultures had a lower proportion of neurons throughout the observation period and in particular at DIV15 (around 55%). We showed that this was due to the increased ratio of non-neuronal cells, by using specific markers for astrocytes and microglia. These results are comparable to postnatal (P0–3) rodent cultures, where the percentage of glia can vary roughly between 10% and 20% (Beaudoin et al., 2012; Todd et al., 2013; Ulloa Severino et al., 2016). The morphology of *M. domestica*-derived astrocytes was strikingly similar to primary rat astrocytes, cultured using similar experimental protocols (Verstraelen et al., 2014; Pozzi et al., 2017; Wolfes et al., 2017). The response of opossum-derived astrocytes to specific growth factors or 3D materials (Puschmann et al., 2014; Ulloa Severino et al., 2016) could be investigated in the future.

### RGCs

RGCs are highly dynamic cells that are actively involved in cortical histogenesis, representing the main progenitor population of all CNS cell lineages (Malatesta et al., 2008; Borrell and Götz, 2014). We have successfully obtained and propagated primary RGCs cultures derived from P3 to P5 opossums that supported both RGCs and neuronal survival *in vitro*. RGCs can be passaged and replated several times and for several weeks or even months, confirming both the neurogenic and gliogenic *in vitro* potential of RGCs. *M. domestica*-derived RGCs can therefore be used as a source of mammalian neurons, glia, and progenitor cells.

### Neuronal Connections and 3D Organoid-Like Structures in Opossum Cortical Cultures

We have shown that primary *M. domestica* cortical neurons follow events and dynamics involved in the formation of functional neuronal networks, very similar to the well-known rodent model. These events involve polarization (i.e., formation of GCs, axon, and dendrite specification; Dotti et al., 1988) and expression of synaptic, as well as excitatory or inhibitory neuronal subtypes markers. We have previously used synapsin, vGLUT2, and GAD65 on the opossum spinal cord (Petrovic et al., 2019), and here we additionally confirmed their expression in developing *M. domestica* primary cortical cultures.

Interestingly, in long-term (DIV30) cultures, the formation of 3D- and organoid-like structures (Supplementary Figure 3) opens the possibility to establish more developed neural systems that could more closely mimic *in vivo* complexity (Todd et al., 2013).

The high degree of protein sequence homology (Mikkelsen et al., 2007) between *M. domestica* and other mammalian species, especially human proteins, should allow, facilitate and encourage neuroscientists for future investigations on opossums.

## Data Availability Statement

The original contributions presented in the study are included in the article/Supplementary Material, further inquiries can be directed to the corresponding author.

## Ethics Statement

The animal study was reviewed and approved by Ethical Committee of the Department of Biotechnology of the University of Rijeka.

## Author Contributions

JB and MM designed and supervised the research. AP, IT, MP, MI, and SM prepared primary cultures. AP, JB, MP, MI, and SM performed immunofluorescence and imaging. AP, JB, MP, and MI analyzed the data. All authors contributed to the article and approved the submitted version.

## Conflict of Interest

The authors declare that the research was conducted in the absence of any commercial or financial relationships that could be construed as a potential conflict of interest.

## Footnotes

^1^http://www.uniprot.org

^2^http://rsbweb.nih.gov/ij/
