# Supplementary material for: Establishment of Long-Term Primary Cortical Neuronal Cultures From Neonatal Opossum Monodelphis domestica
Source: Front Cell Neurosci. 2021 Mar 18;15:661492. doi: 10.3389/fncel.2021.661492 (PMC8012671; doi:10.3389/fncel.2021.661492)
Supplement: Supplementary file 1 [file Data_Sheet_1.docx]

Supplementary Material

# Supplementary Figures

**
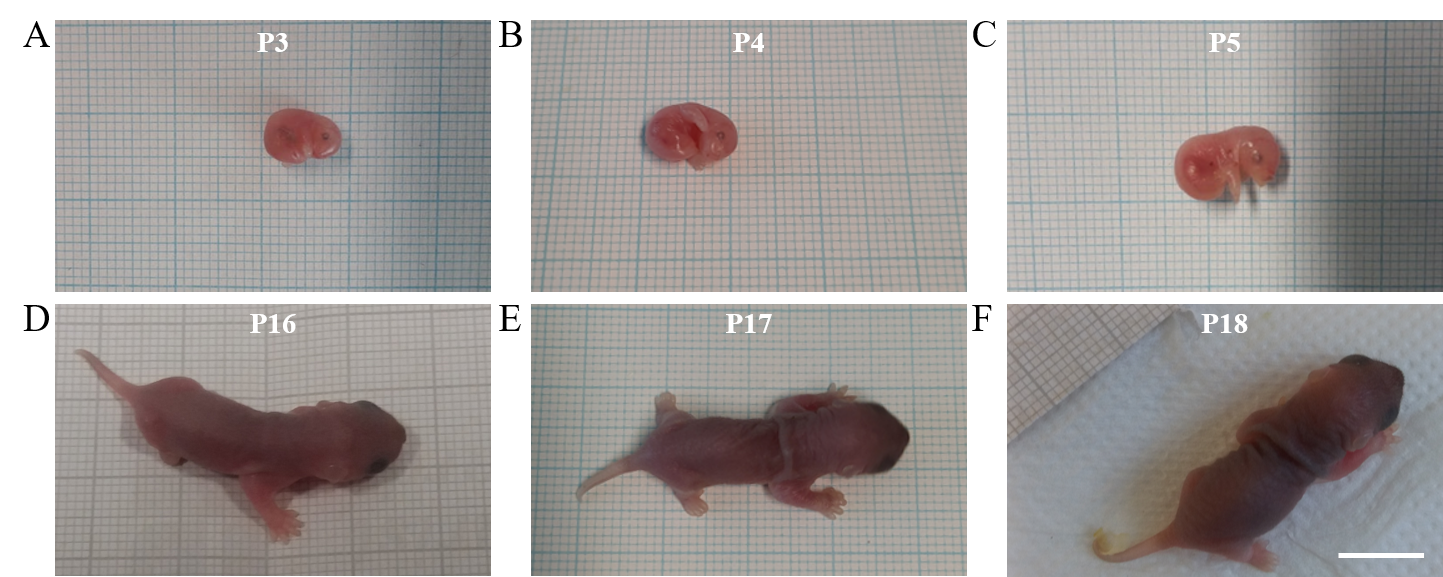
**

**Supplementary Figure 1**. *Monodelphis domestica* at postnatal ages used in this study. **(A)** P3, **(B)** P4, **(C)** P5, **(D)** P16, **(E)** P17, **(F)** P18 opossum pups. Scale bar, 1 cm.


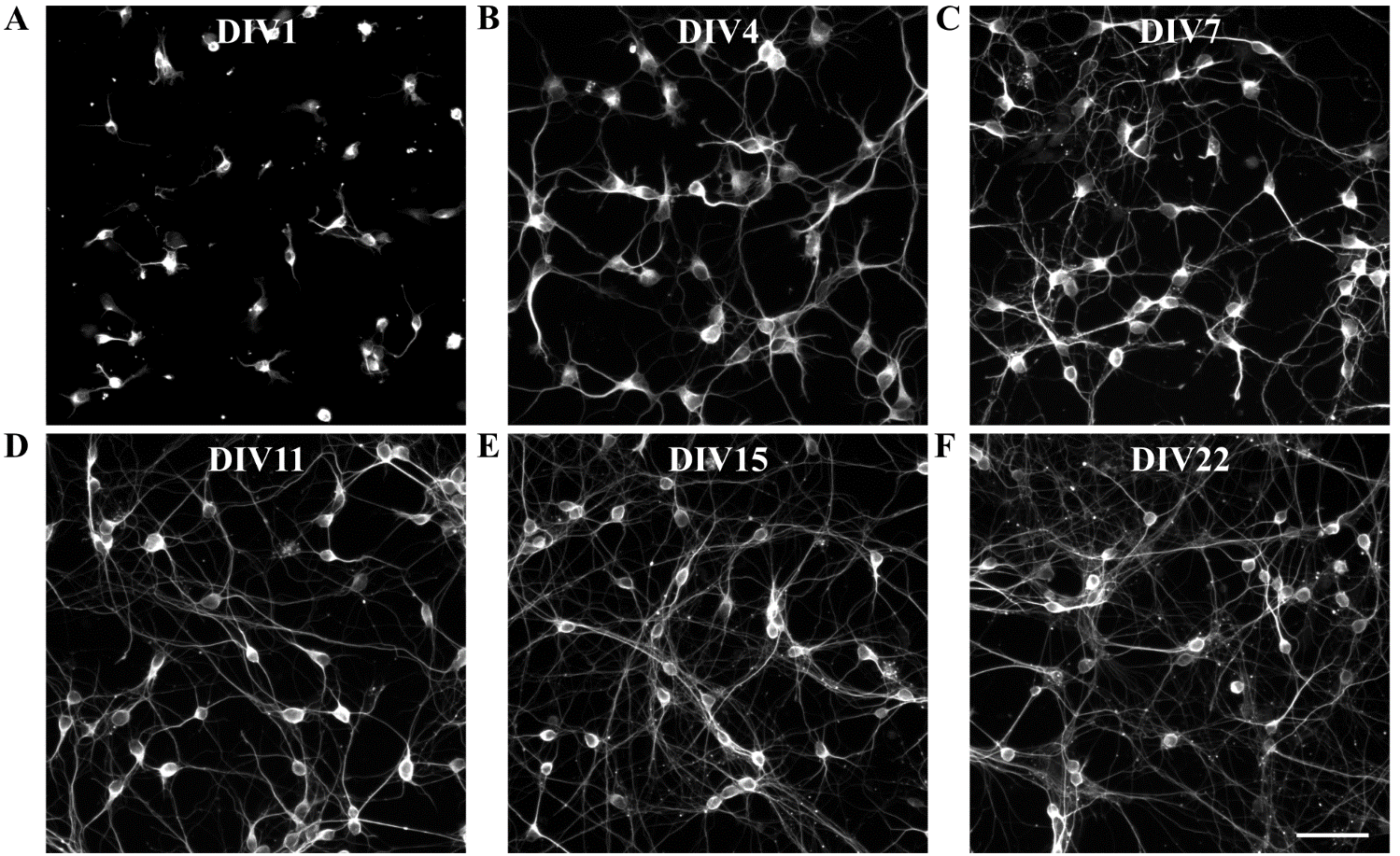


**Supplementary Figure 2**. Primary neuronal cultures derived from P3-5 cortex of *M. domestica.* Cells were fixed and stained for β-tubulin III (TUJ1) at **(A)** DIV1, **(B)** DIV4, **(C)** DIV7, **(D)** DIV11, **(E)** DIV15 and **(F)** DIV22, respectively. Scale bar, 50µm.


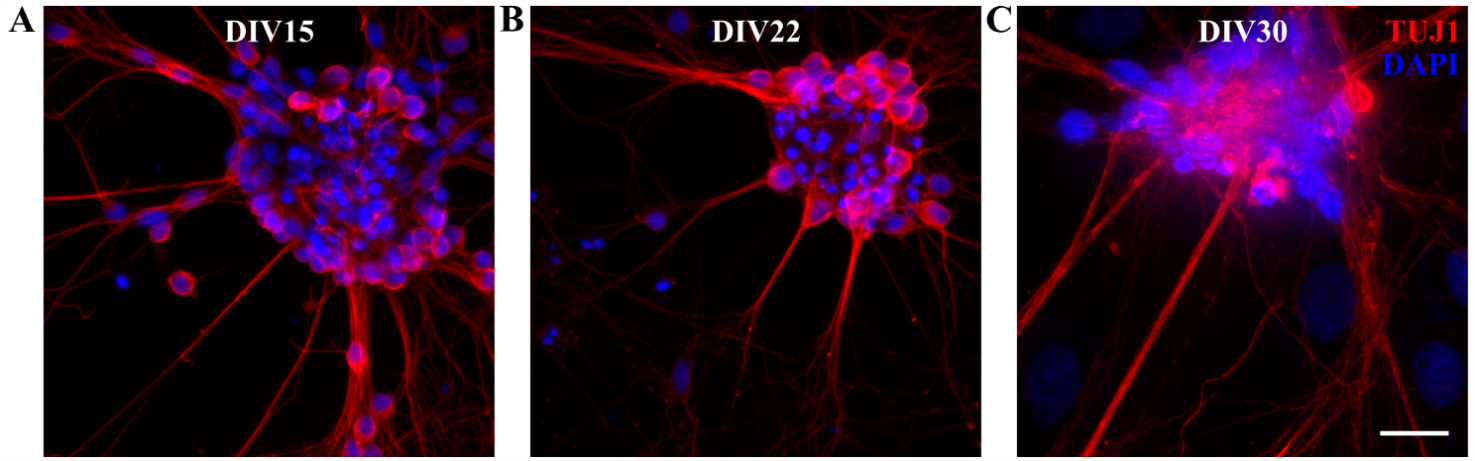


**Supplementary Figure 3**. Formation of cell clusters in long-term cultures. **(A)** DIV15, **(B)** DIV22 and **(C)** DIV30 stained with TUJ1 (red) and DAPI (blue). Scale bar, 25 µm.


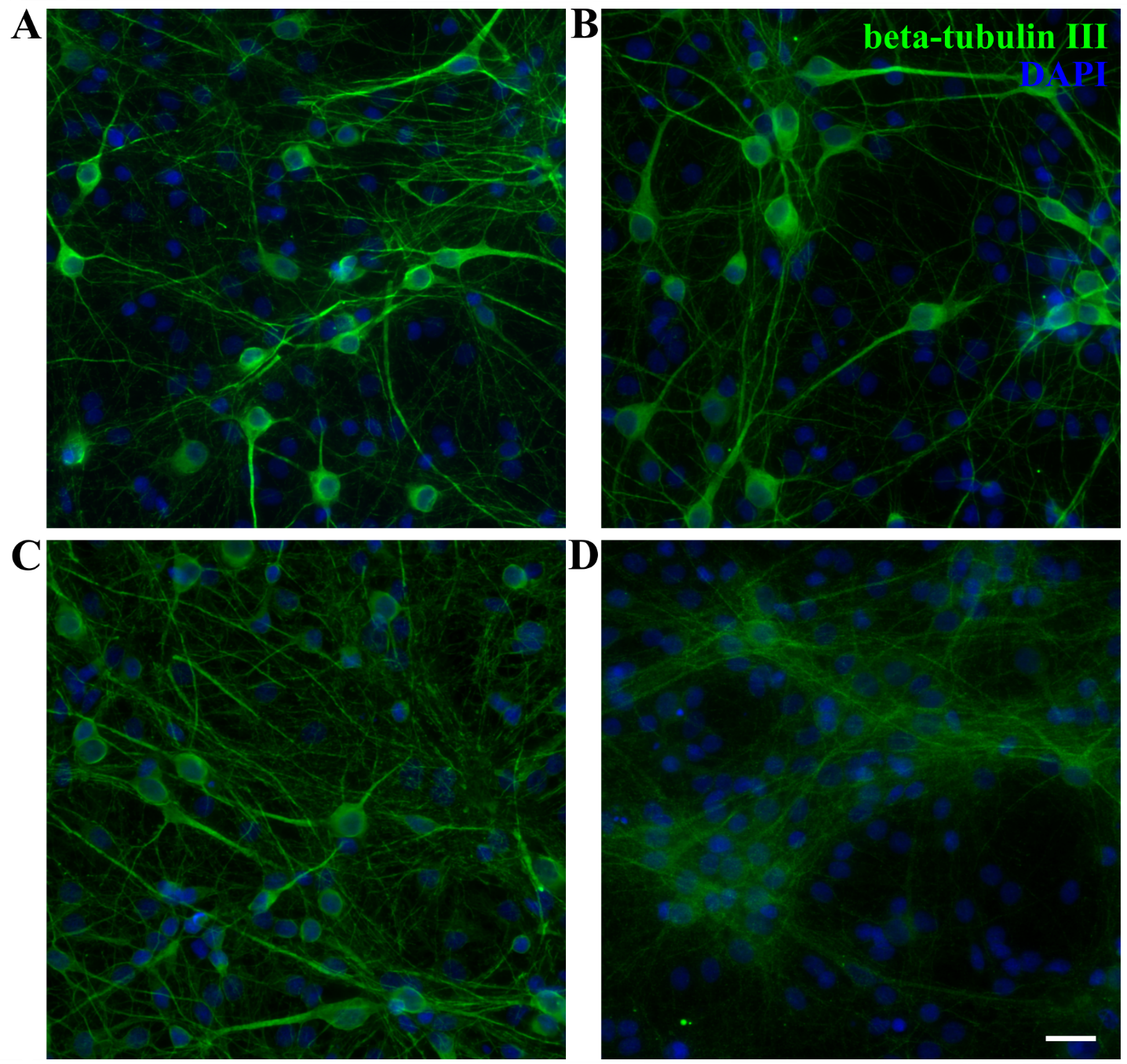


**Supplementary Figure 4.** β-tubulin III immunostaining of neuronal cultures derived from P17 opossum using different primary antibodies. Neuronal cultures were fixed at DIV19 and stained with **(A)** TUJ1, monoclonal IgG_2a_ antibody (Biolegend, Cat# 801201, RRID: AB_2313773, 99.8% Uniprot sequence similarity), **(B)** rabbit polyclonal antibody (Sigma-Aldrich, Cat# T2200, RRID: AB_262133, 90% similarity), **(C)** monoclonal IgG_2a_ antibody (Proteintech, Cat# 66240-1-Ig, RRID: AB_2881629, 99.8% similarity) and **(D)** monoclonal IgG_2b_ antibody (Sigma-Aldrich, Cat# T5076, RRID: AB_532291, 99.8% similarity). Goat anti-mouse (A, C and D) and goat anti-rabbit (B) Alexa Fluor 488 (green) secondary antibodies and DAPI nuclear stain (blue) were used. Scale bar, 25 µm.


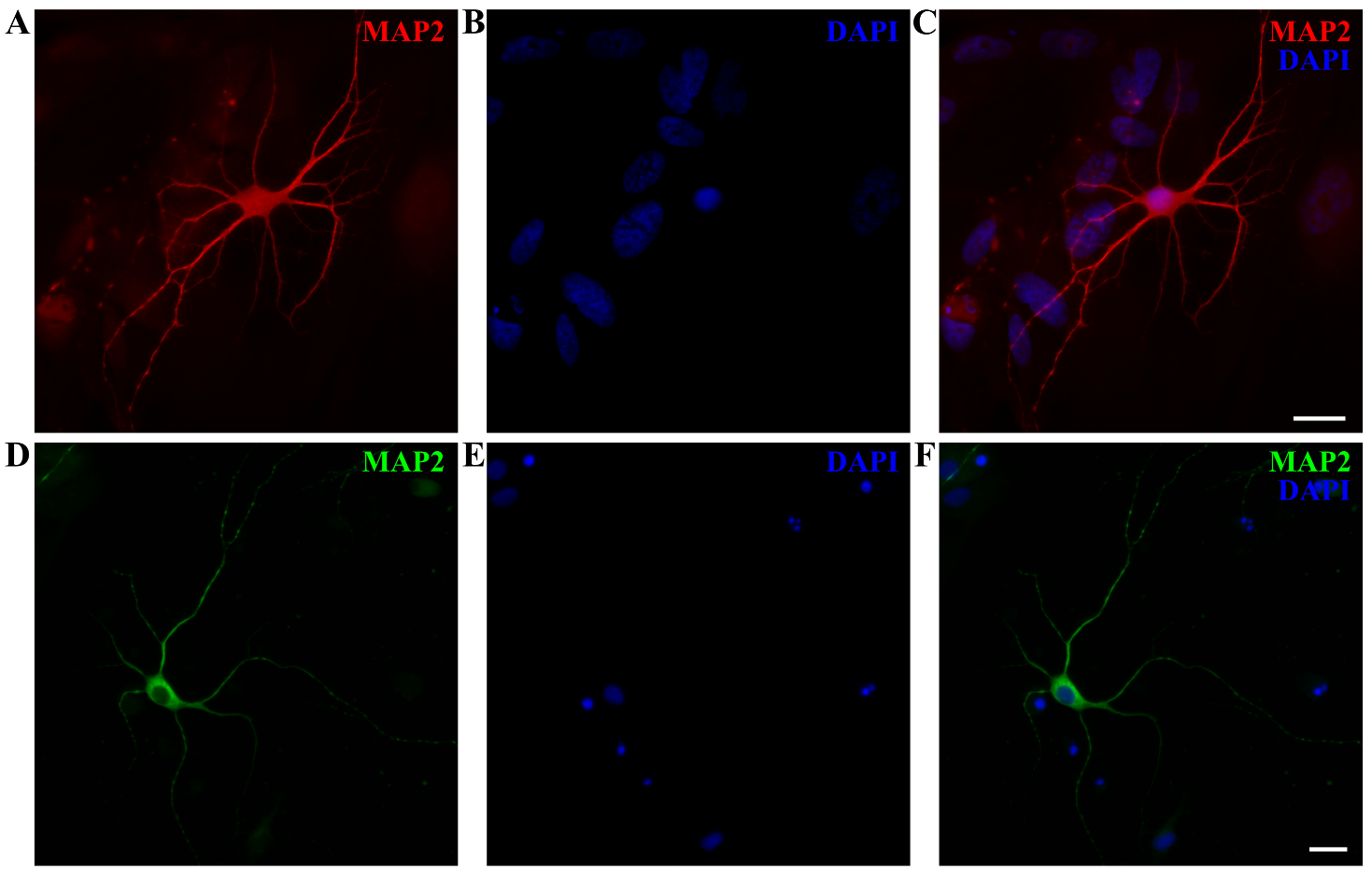


**Supplementary Figure 5.** MAP2 expression in long-term primary cultures. Two different antibodies were utilized: **(A-C)** rabbit polyclonal antibody (Sigma-Aldrich, Cat# M3696, RRID: AB_1840999, red) and (D-F) mouse monoclonal IgG_1_ antibody (Sigma-Aldrich, Cat# M1406, RRID: AB_477171, green) on P3 DIV30 (A-C) and P16 DIV25 (D-F) opossum cortical cultures, respectively. Scale bar, 20 µm.


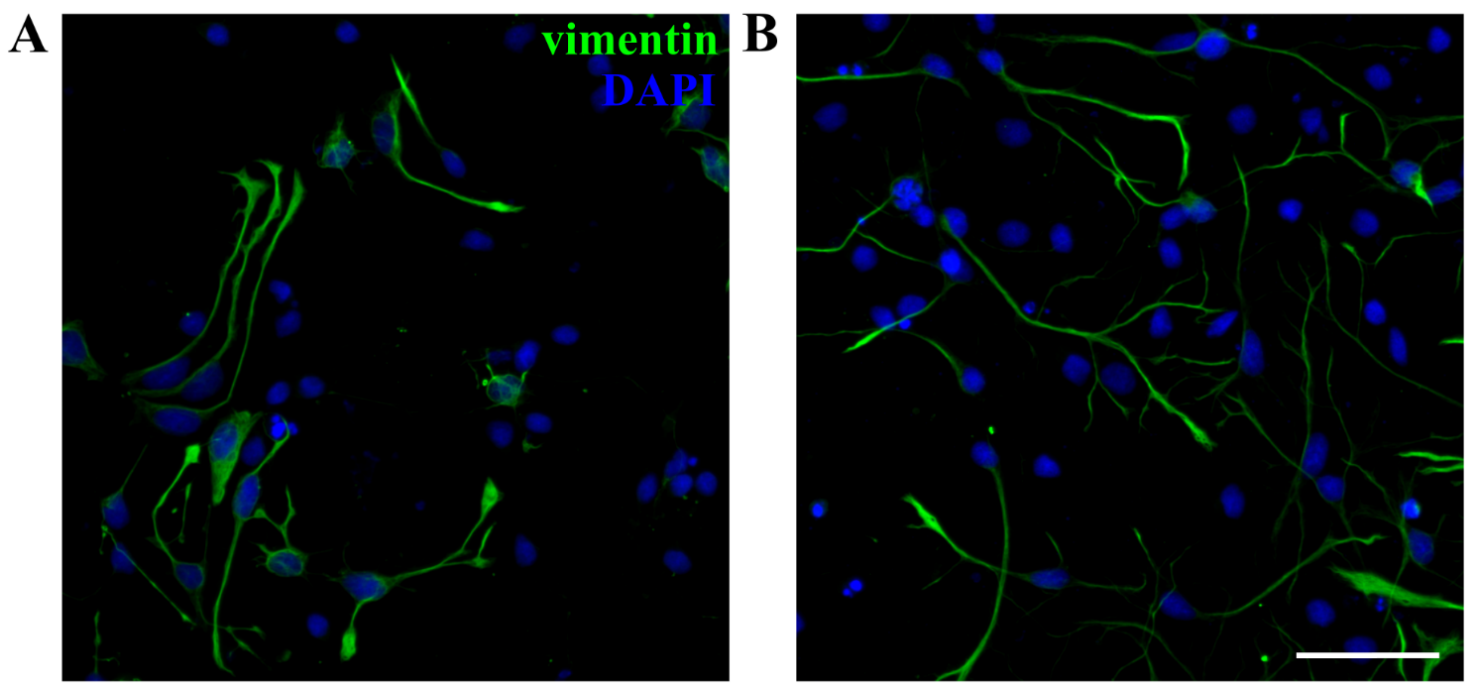


**Supplementary Figure 6**. Vimentin expression in primary cortical astrocytes derived from *M. domestica*. **(A)** P4 and **(B)** P17 cortex was used to obtain primary cultures. At DIV7, cells were fixed and stained for vimentin (Abcam, Cat# ab8069, RRID: AB_306239, 91.7% similarity, green) while cell nuclei were stained with DAPI (blue). Scale bar, 50 µm.


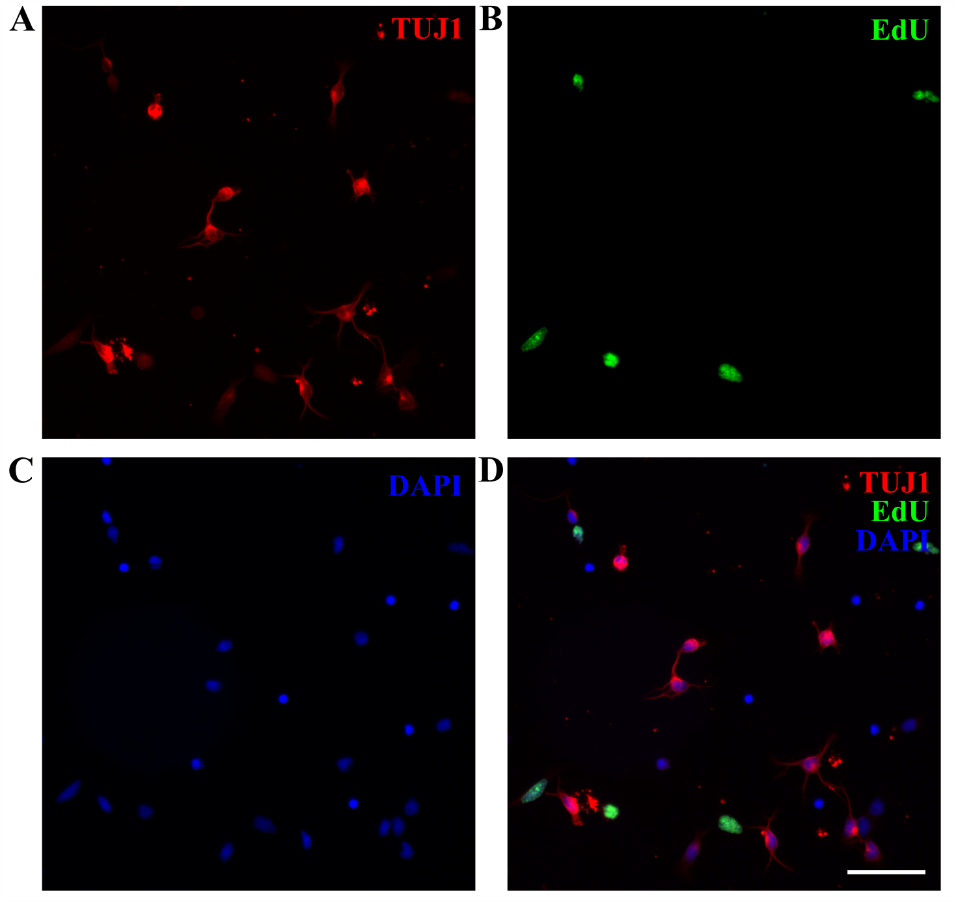


**Supplementary Figure 7**. Proliferative cells and neurons at DIV1. P5 opossum cortex was dissociated and plated in DMEM supplemented with 10% FBS. EdU reagent was added immediately after plating according to producers’ instructions (see Methods). Cells were fixed and stained 24h after plating. **(A)** TUJ1-positive neurons (red), **(B)** EdU-positive proliferative cells (green), **(C)** DAPI nuclear stain (blue) and **(D)** merged image. Scale bar, 50 µm.


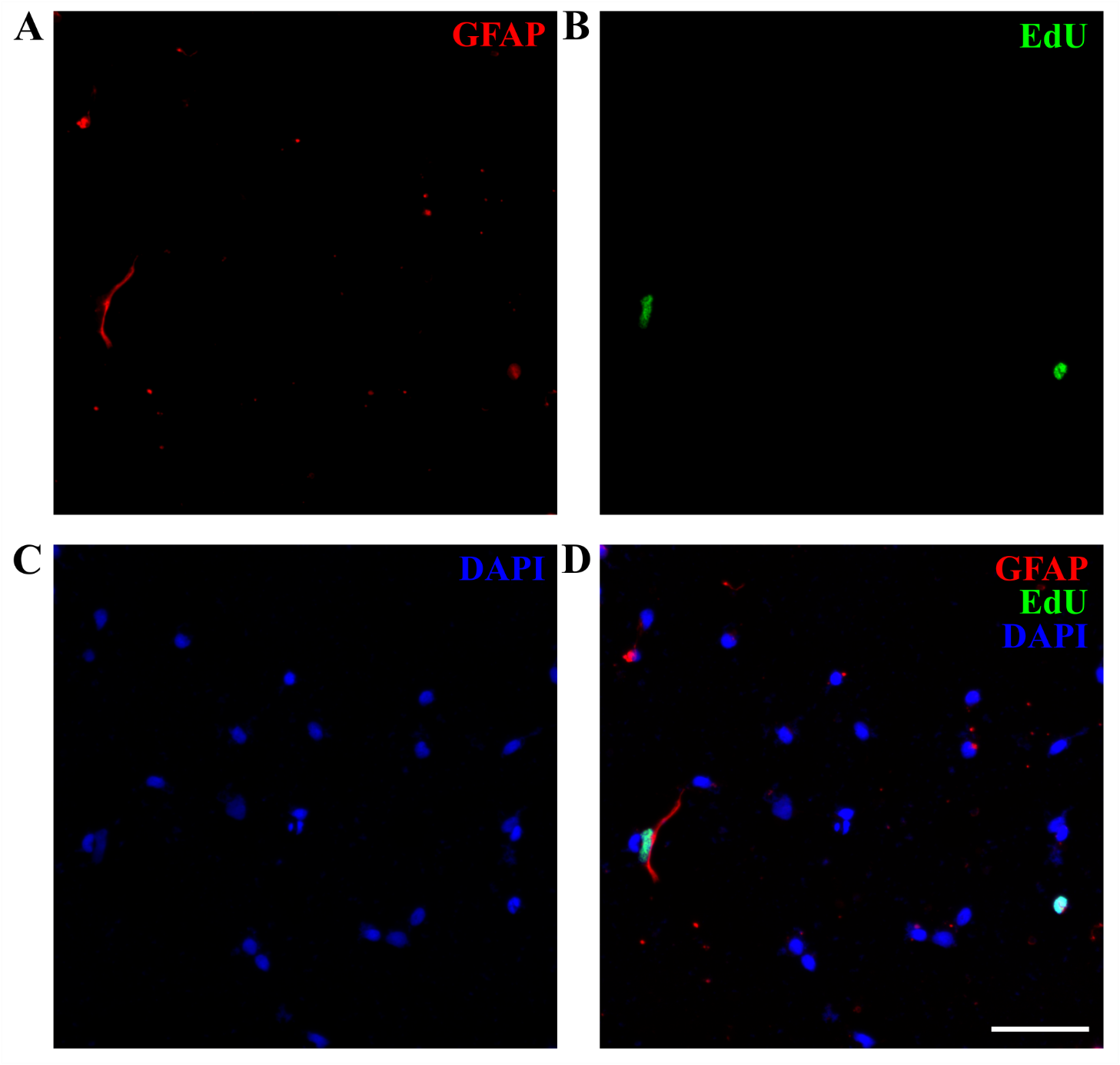


**Supplementary Figure 8**. Proliferative state of RGCs. P5 opossum cortex was dissociated and plated in DMEM supplemented with 10% FBS. EdU reagent was added at DIV1 according to producers’ instructions (see Methods). Cells were fixed and stained at DIV2. **(A)** GFAP-positive (red), **(B)** EdU-positive cells (green), **(C)** DAPI nuclear stain (blue) and **(D)** merged image. Scale bar, 50 µm.
